# Molecular mechanisms of *TTC21B* gene mutations in nephronophthisis type 12 and genetic prevention through PGT

**DOI:** 10.3389/fgene.2025.1710252

**Published:** 2025-11-26

**Authors:** Kai Deng, Jingjing Li, Xitao Hu, Huijuan Wei, Chenyi Wang, Qingqing Cheng, Yu Jiang, Liyi Cai, Di Tang, Guiju Cao, Xiaoyan Wang

**Affiliations:** 1 Department of Cell Biology, School of Basic Medicine, Hebei Medical University, Shijiazhuang, China; 2 Reproductive Medicine Department of Hebei Maternity Hospital, Shijiazhuang, China; 3 Shi Jiazhuang Technology Innovation Center of Precision Prevention and Control of Birth Defects, Shijiazhuang, China; 4 School of Biomedical Engineering, Hubei University of Medicine, Shiyan, China

**Keywords:** nephronophthisis type 12, TTC21B gene, whole-exome sequencing, variant of uncertain significance (VUS), PGT (preimplantation genetic testing), functional validation

## Abstract

**Objective:**

To elucidate the pathogenic mechanism of nephronophthisis type 12 (NPHP12) caused by compound heterozygous mutations in the *TTC21B* gene and to implement preimplantation genetic testing (PGT) for clinical prevention.

**Methods:**

We retrospectively analyzed the clinical data of a pediatric proband with NPHP12. The impact of the identified *TTC21B* mutations (c.895T>C and c.1552T>C) on pre-mRNA splicing, protein structure, and stability was assessed using bioinformatics tools. Functional validation was performed through *in vitro* rescue experiments in renal podocytes, quantifying effects on cellular morphology, ciliogenesis, and ciliary length. For preimplantation genetic testing (PGT), SNP haplotype analysis was employed for embryo selection.

**Results:**

The proband presented with renal failure and compound heterozygous *TTC21B* mutations (paternal c.895T>C [p.C299R] and maternal c.1552T>C [p.C518R]). Bioinformatics suggested potential splicing impacts, but minigene assays did not validate this. While c.895T>C had a minor impact on protein structure, c.1552T>C significantly altered the tertiary structure and stability. *TTC21B* knockdown disrupted podocyte morphology, which was fully rescued by wild-type *TTC21B* but only partially by either mutant. Both mutations impaired ciliogenesis and shortened ciliary length. PGT-M identified a mutation-free embryo (Embryo 1) for transfer, resulting in a healthy live birth.

**Conclusion:**

The compound heterozygous mutations c.895T>C (p.C299R) and c.1552T>C (p.C518R) in *TTC21B* contribute to NPHP12 by disrupting ciliogenesis and podocyte morphology. These findings provide targets for PGT-based embryo selection, successfully preventing the familial recurrence of this disease.

## 1 Introduction

Nephronophthisis (NPHP) is an autosomal recessive inherited nephropathy primarily characterized by tubular interstitial damage, with associated phenotypes including tubular basement membrane destruction, cystic lesions, and interstitial fibrosis, ultimately leading to end-stage renal disease (Alizadeh et al., 2020; Abo and Al-Fiky, 2020). It is also one of the most common causes of renal failure in children and adolescents. Early-stage NPHP may present with atypical renal manifestations such as polydipsia and polyuria. Clinical manifestations typically emerge during the advanced chronic kidney disease (CKD) stage. Diagnostic imaging reveals increased renal parenchymal echogenicity with unclear medullary boundaries, often accompanied by renal cysts or parenchymal atrophy (Chen et al., 2024). Due to the insidious onset and nonspecific early clinical symptoms of this disease, genetic testing has emerged as a critical diagnostic tool (Li et al., 2024). Therefore, elucidating the genotype-phenotype correlations in NPHP is of significant importance for both understanding its pathogenesis and enabling early diagnosis.

The pathogenesis of NPHP is closely associated with ciliary dysfunction (Ben-Yosef et al., 2021), representing a significant subtype of ciliopathies affecting renal podocytes. Renal podocyte cilia are primary cilia, where the intraflagellar transport protein complexes A (IFT-A) and B (IFT-B) together constitute the IFT complex (Klena and Pigino, 2022). This complex is responsible for bidirectional cargo transport along the microtubule axoneme (Lacey et al., 2024). Dysfunction of these cilia leads to a clinical syndrome characterized by chronic tubulointerstitial lesions and extrarenal manifestations (Jacobs et al., 2016). More than 20 pathogenic genes have been identified to be associated with NPHP pathogenesis (Petzold et al., 2023). Proteins encoded by these genes are primarily localized to the transition zone and basal body of cilia, participating in the bidirectional transport of substances between the ciliary interior and exterior (Afzelius, 2004).

Based on the pathogenic genes involved, NPHP can be classified into multiple subtypes, with NPHP1 (caused by *NPHP1* gene mutations), NPHP7 (caused by *GLIS2* gene mutations), and NPHP12 (caused by *TTC21B* gene mutations) (Schueler et al., 2016) are the relatively prevalent subtypes. The *TTC21B* gene, which causes NPHP12, is located on chromosome 2q24.3, containing 29 exons and encoding the intraflagellar transport protein IFT139 (Intraflagellar Transport Protein 139). In primary cilia, IFT139 serves as a key component of the IFT-A complex, participating in the regulation of retrograde transport within cilia (Niwa, 2016).

Here, we report a family with a rare genetic disorder of NPHP12, in which the proband was identified to carry compound heterozygous mutations in the *TTC21B* gene: c.1552T>C (p.C518R) and c.895T>C (p.C299R). Through comprehensive analysis of clinical diagnostic and therapeutic data, we systematically delineated genotype-phenotype associations in NPHP12. Subsequently, bioinformatics analysis was performed to assess the impact of these compound heterozygous mutations on splicing function, mRNA, and protein expression levels. The impact of mutations on pre-mRNA splicing was confirmed by *in vitro* minigene assays. We demonstrated the effects of *TTC21B* mutations on podocyte morphology, ciliogenesis, and ciliary length by immunofluorescence. These findings provide theoretical support for exploring the pathogenicity of *TTC21B* compound heterozygous mutations in NPHP12.

## 2 Materials and methods

### 2.1 Study subjects

In 2008, a full-term vaginal delivery resulted in a female infant. The disease onset was insidious, with renal failure and stage II hypertension discovered at age 5, accompanied by situs inversus and brachydactyly. The condition rapidly progressed to end-stage renal disease, and the patient died of heart failure after 4 months of treatment. In 2011, another full-term vaginal delivery produced a male proband. Renal failure was diagnosed at 5 years and 8 months of age. In January 2017, genetic testing at the Reproductive Medicine Center of Shiyan Renmin Hospital in Hubei Province identified compound heterozygous mutations in the *TTC21B* gene: c.1552T>C (p.C518R) inherited from the mother and c.895T>C (p.C299R) inherited from the father.

### 2.2 Bioinformatics prediction

HSF (https://hsf.genomnis.com/login),SpliceAI(https://spliceailookup.broadinstitute.org), and CBS (https://services.healthtech.dtu.dk/services/NetGene2-2.42/) databases were used to analyze the impact of mutations on pre-mRNA splicing. The SOPMA (https://npsa-prabi.ibcp.fr/cgi-bin/npsa_automat.pl?page=npsa_sopma.html) database, SWISS-MODEL modeling, PyMOL (The PyMOL Molecular Graphics System, Version 2.0) software, Missense3D (http://missense3d.bc.ic.ac.uk/∼missense3d/) database, and DUET (http://biosig.unimelb.edu.au/duet/stability) database were employed to predict the effects of mutations on the secondary and tertiary structures and stability of the protein.

### 2.3 Construction of knockdown cell models

Table 1 lists the target sequences used. The plko.1 vector plasmid was provided by BioEagle Biotechnology Co. (Wuhan). The constructed shRNA was transiently transfected into MPC-5 cells according to the Lipo2000 transfection protocol. Cells were harvested 48 h post-transfection, and knockdown efficiency was validated by qRT-PCR and Western blot assays.

**TABLE 1:** Knockdown target sequence.

Name	Sequence
*TTC21B*-sh1	GCA​CAC​TGA​TGG​AGG​GTA​AAG
*TTC21B*-sh2	CGG​GAG​TTT​GAG​GCC​ATT​AAA
*TTC21B*-sh3	GCC​TTG​GAA​TCC​TAT​TGA​AGC

### 2.4 Analysis of the impact of mutations on *TTC21B* mRNA and protein expression levels

The constructed *TTC21B*-wt, *TTC21B*-mut1, and *TTC21B*-mut2 expression vectors were transfected into HEK293T cells. After 48 h of transfection, total RNA was extracted using the Trizol method, followed by DNA digestion and cDNA synthesis. The mRNA expression levels of *TTC21B* were detected by qRT-PCR. Meanwhile, total cellular proteins were extracted using RIPA lysis buffer, and protein concentrations were measured using a BSA assay kit. After protein denaturation, equal amounts of total protein were subjected to SDS-PAGE electrophoresis, and Western blot was performed to detect the expression levels of *TTC21B* protein.

### 2.5 Immunofluorescence

Mouse podocytes (MPC-5) and transfected cells (plKO.1, plKO.1-sh*TTC21B*, plKO.1-sh*TTC21B*+wt, plKO.1-sh*TTC21B*+mut1, plKO.1-sh*TTC21B*+mut2) were seeded onto pre-treated coverslips in culture plates. After 12 h of cell culture, cells were fixed with 4% paraformaldehyde. Following PBS washing and Triton X-100 permeabilization, cells were blocked with 5% BSA for 1 h. After incubation, cells were washed 3 times with PBS and then incubated with mouse anti-human *TTC21B* primary antibody diluted in 1% BSA at 4 °C overnight. The next day, cells were washed 3 times with PBS and incubated with CoraLite488-conjugated Goat Anti-Mouse IgG(H+L) secondary antibody diluted in 1% BSA in a humidified chamber for 1 h. Finally, one drop of DAPI mounting solution (0.5 μg/mL) was added for sealing, and cellular morphological changes were examined under a fluorescence microscope.

### 2.6 Ciliary morphology observation

HEK293T cells with ciliary structures were cultured in DMEM medium containing 10% fetal bovine serum. Appropriate HEK293T cells were seeded on coverslips and induced for ciliogenesis by serum starvation for 48 h. Following fixation with 4% paraformaldehyde and permeabilization with 1% Triton-X-100, cells were blocked with 2% bovine serum albumin (BSA) for 1 h. They were then incubated with cilia-specific CoraLite® Plus 488-conjugated ARL13B monoclonal antibody at room temperature for 1 h. After adding one drop of DAPI mounting solution (0.5 μg/mL) for sealing, cellular morphological changes were examined under a fluorescence microscope. Ciliary structures were observed under a fluorescence microscope, and images were processed using ImageJ software. Three images were evaluated per experiment. The percentage of ciliated cells over the total cell count was calculated, and the average ciliary length was statistically compared between different groups. Results were expressed as Mean ± standard deviation.

### 2.7 Preimplantation genetic testing (PGT) and embryo selection

Indications for PGT-M: Families with a clearly pathogenic or likely pathogenic gene variant and well-defined disease-linked markers, where the severity of the disease and the family’s specific circumstances are carefully considered, and informed consent was obtained from the couple.

After identifying the pathogenic gene variants and their origins through genetic testing, the haplotypes were reconstructed using linkage analysis and the maximum likelihood method, and were applied in PGT-M (pre-implantation genetic testing for embryos) to accurately identify embryos carrying specific genetic variations. Based on the analysis results, further embryo culture and blastocyst biopsy were conducted. The embryos were cultured until day 5 (blastocyst stage), and 5–8 trophectoderm cells were collected for genetic analysis to determine whether the embryos exhibit chromosomal numerical abnormalities or single-gene disorders. Normal embryos were selected for uterine implantation to achieve a normal pregnancy. After delivery, postnatal genetic verification was performed on the newborn.

## 3 Results

### 3.1 Clinical phenotype and pedigree analysis

**FIGURE 1:**
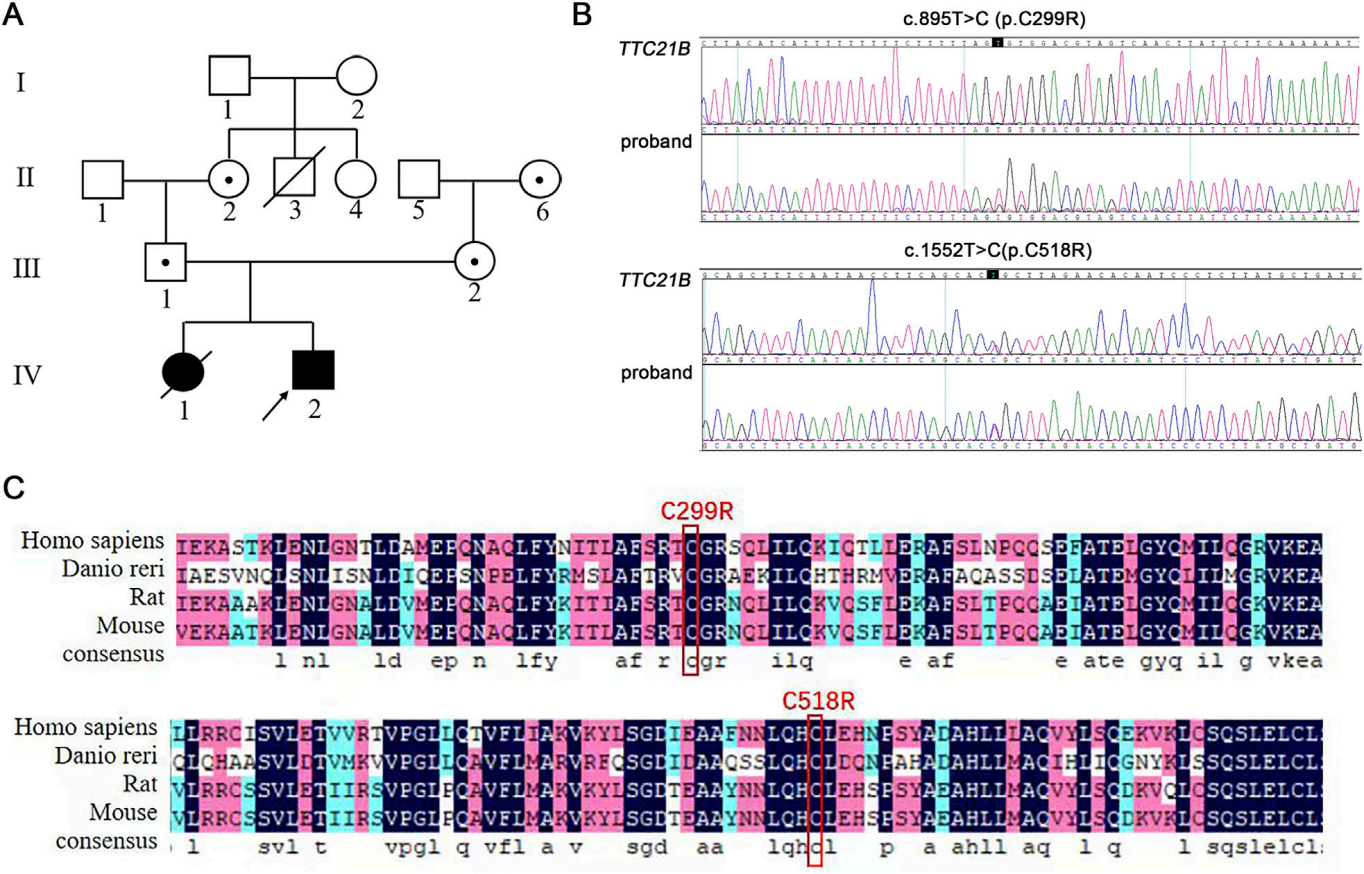
Pedigree analysis and Sanger sequencing results of the proband. **(A)** The family pedigree of patient with mutated *TTC21B*. **(B)** Sanger sequencing results of the proband. **(C)** Analysis of amino acid conservation at the mutation site.

The proband’s mother sought medical attention due to a history of multiple embryonic arrests and death of infant from renal failure. She married in 2005 and underwent a curettage procedure at 3 months of pregnancy due to embryonic arrest. In 2007, she underwent an induced abortion and curettage at 4 months of pregnancy due to fetal death. In 2008, she delivered a full-term baby girl vaginally. At age five, the child was diagnosed with renal failure. After 4 months of treatment, she died from heart failure. In 2011, she delivered a full-term male child who also developed renal failure at 5 years old. Chromosomal examination in January 2017 revealed compound heterozygous mutations in the *TTC21B* gene (c.1552T>C [p.C518R] and c.895T>C [p.C299R]). Maternal genetic testing revealed a heterozygous mutation in the *TTC21B* gene at position c.1552T>C, while paternal genetic testing indicated a heterozygous mutation in the *TTC21B* gene at position c.895T>C. In February 2019, she underwent hysteroscopy at Shiyan Renmin Hospital, which revealed normal uterine morphology, and requested preimplantation genetic diagnosis. Over the past months, her mental status, appetite, and sleep were normal, with regular bowel and bladder function, and no significant changes in physical strength or weight. There was no family history of genetic diseases. Both children exhibited identical renal failure symptoms, and the proband, mother, and father all tested positive for *TTC21B* gene mutations, suggesting an autosomal recessive inheritance pattern. Based on the proband’s genetic test results, *TTC21B* was likely the pathogenic gene, with an autosomal recessive inheritance mode (Figure 1A).

The proband was identified to carry compound heterozygous mutations in *TTC21B*: c.895T>C and c.1552T>C (Figure 1B). Among these, the c.895T>C mutation had not been previously documented in databases. Both c.1552T>C (p.C518R) and c.895T>C (p.C299R) mutations occurred at cysteine residues that were highly conserved across four common species (Figure 1C), suggesting potential impacts on protein structure, stability, and function. According to ACMG classification criteria, these mutations carried PM3 (detection of a pathogenic variant in trans for a recessive disorder), PM2_supporting (absence in ESP, 1000 Genomes, and ExAC databases in normal populations), and PP3 (predicted to be deleterious by multiple computational tools), warranting a classification of Variant of Uncertain Significance (VUS). While the c.1552T>C variant had been reported, it was also classified as VUS. Therefore, the pathogenicity of *TTC21B* compound heterozygous mutations required further investigation.

### 3.2 Impact of mutations on Pre-mRNA splicing

#### 3.2.1 Bioinformatics predictions

Using the HSF, SpliceAI, and CBS databases, we predicted the potential impact of the c.1552T>C and c.895T>C mutations on *TTC21B* pre-mRNA splicing. The c.1552T>C mutation was predicted by both HSF and SpliceAI to likely have no impact on splicing, whereas CBS analysis suggested that this mutation might alter the original acceptor splice site, potentially affecting splicing efficiency. For c.895T>C, SpliceAI and HSF predicted no effect, while CBS indicated a potential impact (Table 2).

**TABLE 2:** Bioinformatics predictions.

Gene	Mutation location	SpliceAI	CBS	HSF	gnomAD	Mutation taster
*TTC21B*	c.895T>C	Not affect	Affect	Not affect	Not included	Deleterious
*TTC21B*	c.1552T>C	Not affect	Affect	Not affect	Included	Deleterious

#### 3.2.2 Minigene assay results

To experimentally validate these predictions, we performed *in vitro* minigene assays in Hela and HEK293T cells. The results demonstrated that neither the c.895T>C nor the c.1552T>C mutation altered the normal splicing pattern of *TTC21B* pre-mRNA (Supplementary Figure S1), indicating that aberrant splicing is not the primary pathogenic mechanism for these variants.

### 3.3 Impact of mutations on protein structure and stability

**FIGURE 2:**
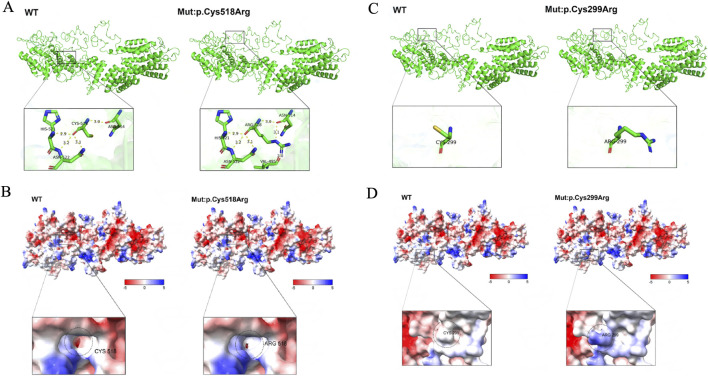
The influence of mutations on the tertiary structure of proteins and the surface electrostatic potential. **(A)** The impact of mutation p.Cys518Arg on the protein’s tertiary structure. **(B)** The influence of mutation p.Cys518Arg on the protein’s surface electrostatic potential. **(C)** The effect of mutation p.Cys299Arg on the protein’s tertiary structure. **(D)** The impact of mutation p.Cys299Arg on the protein’s surface electrostatic potential.

Through SWISS-MODEL modeling and PyMOL analysis, it was found that after the c.895T>C mutation, the main chain CYS299 did not form new hydrogen bonds (Figure 2C), suggesting that the mutation may not affect the main chain and side chain structure of *TTC21B*. However, PyMOL’s Adaptive Poisson-Boltzmann Solver (APBS) analysis showed that the protein surface electrostatic potential shifted from neutral to positive polarity after the mutation (Figure 2D), indicating that the mutation would affect the surface electrostatic potential of *TTC21B* but would not significantly impact its tertiary structure. Additionally, Missense3D prediction results showed that the mutation had no destructive effect on the tertiary structure. Moreover, the predicted protein stability changes (ΔΔG) results from mCSM, SDM, and DUET indicated that the mutation would not alter the stability of the protein structure. These findings suggest that mutation c.895T>C had minimal impact on the tertiary structure and stability of the protein.

The same prediction method was applied, revealing that after the *TTC21B* c.1552T>C mutation, the hydrogen bond distances between the main chain CYS518 and ASN514, HIS521, and ASN522 showed no significant changes (Table 3), suggesting that the mutation may not affect the main chain structure of *TTC21B*. However, new hydrogen bonds formed between the side chain ARG518 and VAL495, as well as between ARG518 and ASN514 (Figure 2A), indicating that the mutation may influence the side chain structure of *TTC21B*. In conclusion, this mutation may alter the tertiary structure of *TTC21B*. APBS analysis in PyMOL showed that the electrostatic potential on the protein surface of *TTC21B* shifted from negative to neutral after the mutation (Figure 2B), suggesting that the mutation affected the molecular surface electrostatic potential of the protein.

**TABLE 3:** Hydrogen bond distances associated with the *TTC21B* c.1552T>C (p.C518R) mutation.

Interaction type	Wild-Type (WT)	Distance (Å)	Mutant (p.C518R)	Distance (Å)
Main chain	CYS518, ASN514	3.0	ARG518, ASN514	3.0
	CYS518, HIS521	2.9	ARG518, HIS521	2.9
	CYS518, ASN522	3.2	ARG518, ASN522	3.2
	CYS518, ASN522	3.1	ARG518, ASN522	3.1
Side chain			ARG518, VAL495	2.0
			ARG518, ASN514	3.1

### 3.4 TTC21B mutations disrupt podocyte morphology

**FIGURE 3:**
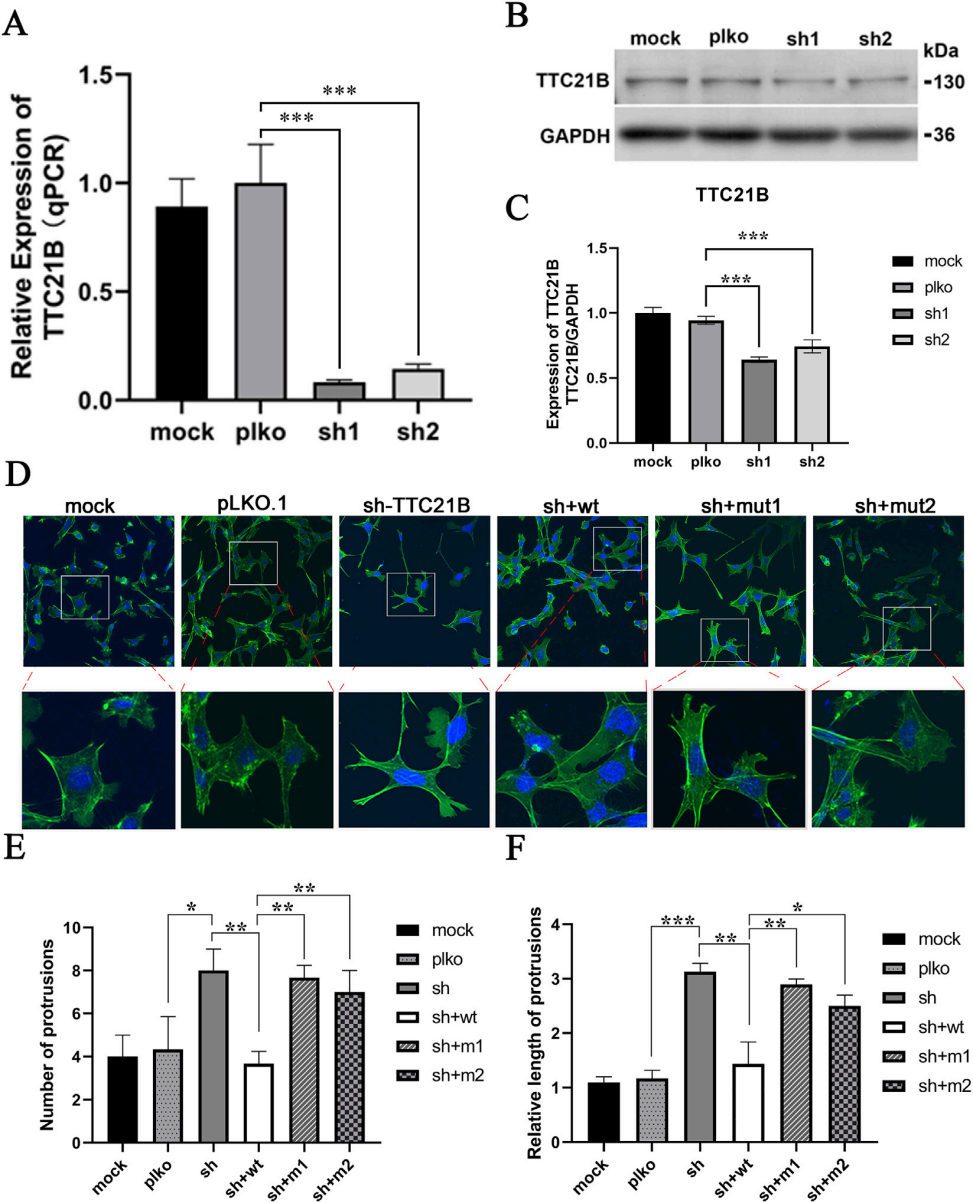
The effects of mutations on the morphology of podocytes. **(A)** qRT-PCR verification of the knockdown effect of *TTC21B*. **(B)** Western blot verification of the knockdown effect of *TTC21B*. **(C)** The gray-scale statistical results of Western blot. **(D)** The effect of mutations on the morphology of podocytes. **(E)** The statistics of the number of protrusions. **(F)** Statistics of protrusion lengths.

A stable TTC21B knockdown cell line was constructed using lentiviral infection. qRT-PCR and Western blot confirmed a significant decrease in TTC21B mRNA and protein expression levels (Figures 3A–C). Immunofluorescence experiments showed that in the sh-TTC21B group, podocyte foot processes became elongated with reduced intercellular connections. Complementation with wild-type TTC21B restored most foot processes to the wild-type state, whereas complementation with mut1 (c.895T>C) or mut2 (c.1552T>C) only partially restored foot process morphology, with irregular shapes and less tight intercellular connections (Figure 3D). Quantification confirmed that the number and length of foot processes in mut1- and mut2-rescued cells were significantly lower than in wild-type-rescued cells (Figures 3E,F).

### 3.5 TTC21B mutations impair ciliogenesis and shorten ciliary length

**FIGURE 4:**
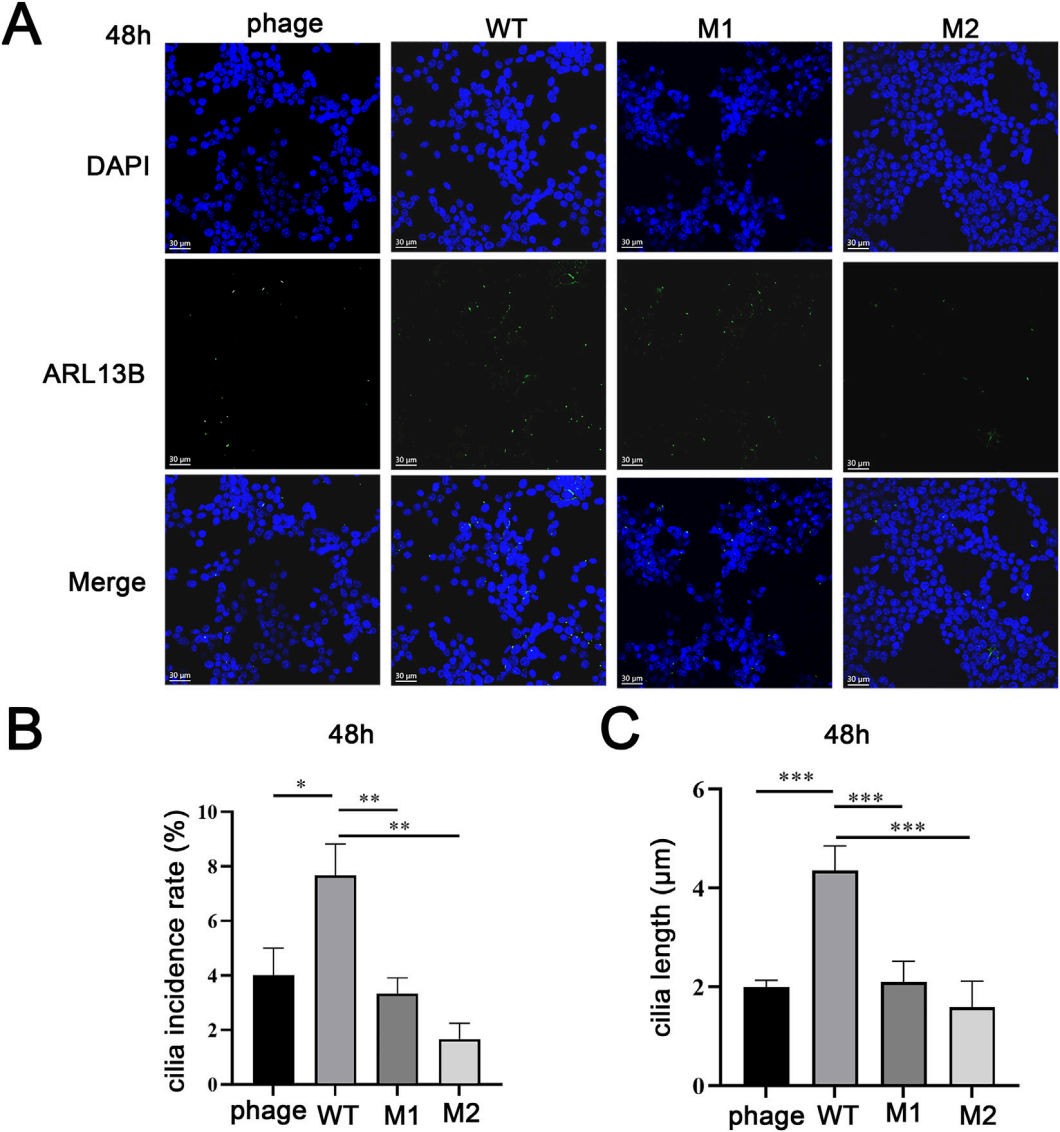
The effects of mutations on the cilia. **(A)** The impact of mutations on cilia formation. **(B)** Comparison of cilia formation rate. **(C)** Average length comparison of cilia.

Using ARL13B to label cilia, we assessed ciliogenesis and ciliary length after serum starvation (Figure 4A). The proportion of ciliated cells in the wild-type group was significantly higher than in the empty vector group, while both mut1 and mut2 showed significantly lower proportions compared to wild-type (P < 0.05) (Figure 4B). Similarly, the average ciliary length in the wild-type group was significantly longer than in the empty vector group, while both mutants exhibited significantly shorter cilia (P < 0.05) (Figure 4C). No significant differences were observed between mut1 and mut2 in either ciliated cell proportion or ciliary length.

### 3.6 PGT technology blocks familial inheritance

According to the ACMG/AMP guidelines, the strength of the PS3 evidence code is based on the quality and conclusiveness of the functional data. Our robust cellular phenotype data—demonstrating partial rescue of podocyte morphology and defects in ciliogenesis—provide PS3 (Moderate) level evidence, as these assays convincingly show a damaging effect of the mutations. Combined with other evidence (PM3, PM2_supporting, PP3), the variants were upgraded to Likely Pathogenic, meeting the clinical indications for PGT-M.

**FIGURE 5:**
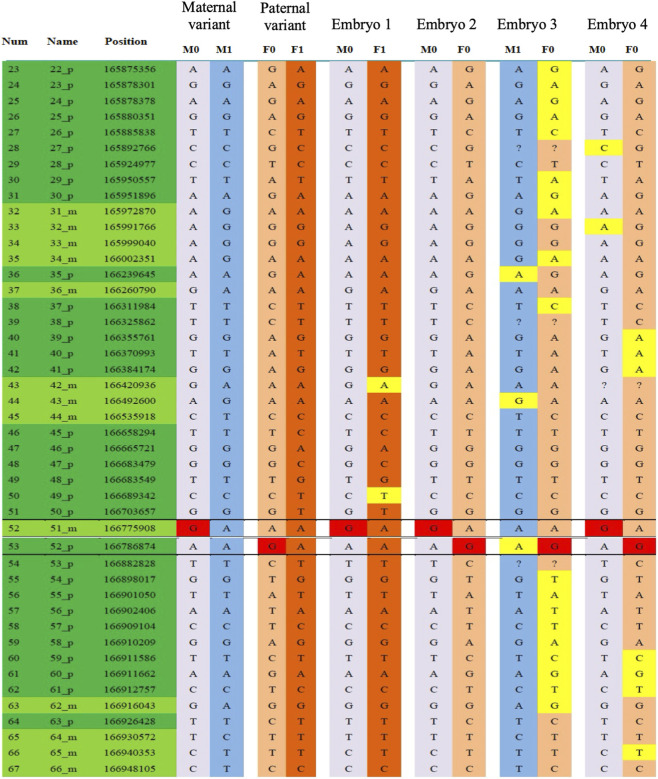
PGT-assisted conception. SNP haplotype results as part of PGT-M analysis.

The embryo screening results after PGT-M showed that embryos No. 2 and No. 4 were compound heterozygous mutations, classified as pathogenic embryos; embryos No. 1 and No. 3 were heterozygous carriers with normal phenotypes. Chromosomal CNV analysis revealed that embryo No. 3 was missing one copy of chromosome 21, Chromosomal CNV analysis revealed that embryo No. 3 exhibited monosomy 21, while embryo No. 1 was euploid and thus eligible for transplantation (Figure 5). After transplantation of embryo No. 1, prenatal diagnosis was declined during pregnancy due to low placental placement. In May 2020, a healthy male infant was born, and genetic validation confirmed maternal carrier status.

## 4 Discussion

This study reported a case of NPHP12-type disease caused by *TTC21B* compound heterozygous mutation, with the proband exhibiting renal failure clinical phenotype. A review of past medical history revealed that a female infant in the family had presented with moderate proteinuria, renal impairment, stage II hypertension, accompanied by situs inversus and brachydactyly, rapidly progressing to end-stage renal disease and ultimately succumbing. Genetic testing was not performed on this female infant. The proband was a later-born male infant who developed renal failure at 5 years of age, with compound heterozygous mutations in *TTC21B* gene. The association between *TTC21B* gene mutations and NPHP12-type disease has been reported in multiple literature (Abo and Al-Fiky, 2020; Li et al., 2024; Wang D. et al., 2022). However, the specific mechanisms by which *TTC21B* gene mutations lead to NPHP12-type occurrence remain incompletely elucidated.

To determine the inheritance pattern of this mutation, genetic validation was performed on the father and mother, confirming that the father carried the c.895T>C (p.C299R) mutation and the mother carried the c.1552T>C (p.C518R) mutation. The proband’s compound heterozygous mutations were inherited from each parent, consistent with autosomal recessive inheritance patterns. According to ACMG classification criteria, both c.895T>C (p.C299R) and c.1552T>C (p.C518R) mutations were classified as variants of uncertain significance (VUS), requiring further functional studies to confirm their pathogenicity.

Bioinformatics analysis confirmed that c.1552T>C and c.895T>C occur near splice sites, potentially disrupting cis-splicing regulatory elements and leading to exon skipping or intron retention, ultimately resulting in abnormal transcript production. Abnormal splicing causes partial/complete loss of gene function, ultimately leading to disease pathogenesis (Nikom and Zheng, 2023). Subsequently, minigene assays were performed *in vitro* to analyze the impact of these mutations on pre-mRNA splicing. The results demonstrated that neither c.895T>C nor c.1552T>C affected normal mRNA splicing. This inconsistency between experimental results and bioinformatics predictions may be related to the complexity of splicing regulation (Lee and Rio, 2015).

Since splice-site mutations may alter mRNA coding sequences, they could adversely affect protein structure and stability (Studer et al., 2013). Further analysis through homology modeling revealed that the c.895T>C mutation had a minor impact on protein tertiary structure and stability. The wild-type cysteine at this position did not participate in disulfide bond formation, so the mutation had limited effects on structural stability. In contrast, the c.1552T>C mutation significantly affected protein tertiary structure and stability. This may be related to the following reasons: the wild-type at this position participates in the formation of disulfide bonds, and after the mutation, it was replaced by arginine, preventing the formation of disulfide bonds and thus disrupting the local hydrophobic core and structural stability of the protein (Hait et al., 2020).

NPHP patients exhibit both tubulopathy and glomerulopathy (Wolf et al., 2024), as ciliary defects can simultaneously affect renal tubular and glomerular function. In renal tubular cells, cilia primarily sense tubular fluid flow and mediate signaling (Bai et al., 2022); their dysfunction can lead to tubular atrophy, cyst formation, and electrolyte disturbances. In podocytes (glomeruli), ciliary defects—particularly of primary cilia—may cause foot process effacement and proteinuria by disrupting cytoskeletal dynamics (Ye et al., 2022), extracellular matrix homeostasis, or critical signaling pathways (e.g., Hedgehog pathway). Thus, the tubulopathy and glomerulopathy in NPHP may share a common upstream mechanism rooted in ciliary dysfunction. Previous reports have shown that the *TTC21B* gene is associated with cilium formation (Driver et al., 2017; Davis et al., 2011). Ciliary dysfunction leads to podocyte morphological abnormalities and filtration barrier dysfunction, ultimately causing renal impairment (Gascue et al., 2011). Based on this, this study employed immunofluorescence to observe the impact of *TTC21B* gene mutations on podocyte morphology. The results demonstrated that knockdown of *TTC21B* altered the normal morphology of podocytes, whereas complementation with the wild-type (wt) restored the typical podocyte morphology. However, complementation with mut1 (c.895T>C) and mut2 (c.1552T>C) only partially restored podocyte morphology, suggesting that *TTC21B* gene mutations can affect podocyte morphology. The altered podocyte morphology adversely affected the filtration barrier function, leading to abnormal plasma protein filtration into the urine (Brinkkoetter et al., 2013; Kim et al., 2023), thereby contributing to renal injury.

To investigate the underlying cause of podocyte morphological abnormalities induced by *TTC21B* gene mutations, the impact of the mutation on cilia formation was further observed. The results demonstrated that both mut1 and mut2 reduced cilium formation rates, with the average cilium length significantly shorter than the wild-type. The *TTC21B*-encoded protein IFT139 is a core component of the retrograde transport complex IFT-A (Wang W. et al., 2022). Mutations in this gene can cause the dissociation of the IFT-A complex, impairing its ability to bind retrograde transport cargo, thereby affecting ciliary length regulation (Jiang et al., 2023). The alterations in cilium number and length may impair primary cilium signaling function, affecting pathways such as Wnt and Hedgehog (Liu et al., 2014; Bangs and Anderson, 2017), thereby leading to podocyte morphological changes, reduced filtration surface area, and ultimately contributing to renal dysfunction.

Currently, there is no cure for genetic diseases caused by single-gene mutations such as *TTC21B*. Preventing the birth of affected infants was crucial, and prenatal diagnosis and preimplantation genetic testing (PGT) were the primary prevention strategies (Greco et al., 2020). PGT aims to select embryos with normal or low-risk genetic profiles for transplantation, enabling healthy reproduction. The development of PGT technology has significantly reduced birth defects associated with single-gene disorders. PGT specifically targeting monogenic diseases was termed PGT-M (Parikh et al., 2023). In this study, PGT-M technology was used to screen out Embryo No. 1 for transplantation. Following embryo transfer, a healthy male infant was born in May 2020, with genetic confirmation of maternal carrier status. Follow-up to date has shown the child’s healthy growth without any related clinical symptoms.

To summarize, this study reported a case of renal failure caused by a compound heterozygous mutation in *TTC21B*. Pathogenicity analysis of the mutation confirmed that the compound heterozygous mutations c.895T>C (p.C299R) and c.1552T>C (p.C518R) in the *TTC21B* gene likely contribute to juvenile nephronophthisis type 12 by affecting cilium formation and length, thereby altering podocyte morphology. This study further expanded the understanding of its pathogenic mechanisms, facilitating better identification of disease-clinical phenotype correlations and elucidating the relationship between *TTC21B* gene mutations and various diseases. These findings provided support for early diagnosis and treatment, as well as a basis for genetic counseling and personalized medicine. More importantly, our study establishes a comprehensive and translational research paradigm, transitioning seamlessly from “clinical identification” to “functional elucidation” and ultimately to “clinical prevention.” We began with the clinical presentation of a rare disease in a family, identified VUSs through genetic testing, and subsequently employed a series of *in silico* and *in vitro* functional experiments to provide direct evidence for their pathogenicity, thereby upgrading their ACMG classification. Ultimately, we translated these fundamental research findings into a successful clinical intervention via PGT-M, resulting in the birth of a healthy child and effectively blocking the vertical transmission of the genetic disorder within the family. This closed-loop strategy not only resolves the ambiguity surrounding the *TTC21B* VUSs but also serves as an exemplary model for the diagnosis, management, and prevention of many other rare monogenic diseases, demonstrating the powerful synergy between mechanistic investigation and precision reproductive medicine.

## Data Availability

The original contributions presented in the study are included in the article/Supplementary Material, further inquiries can be directed to the corresponding author.
