# Supplementary material for: Molecular mechanisms of TTC21B gene mutations in nephronophthisis type 12 and genetic prevention through PGT
Source: Front Genet. 2025 Nov 26;16:1710252. doi: 10.3389/fgene.2025.1710252 (PMC12688276; doi:10.3389/fgene.2025.1710252)
Supplement: Supplementary file 1 [file DataSheet1.docx]

Supplementary methods

Minigene assay

The *TTC21B*-wt, *TTC21B*-mut1, and *TTC21B*-mut2 minigene recombinant plasmids were transiently transfected into Hela and HEK293T cell lines, following the transfection protocol provided in the lipid reagent manual. Cells were harvested 48 hours post-transfection. Total RNA was extracted from cell samples using the Trizol method, followed by phenol-chloroform purification. After measuring concentration, assessing purity, and verifying integrity, equal amounts of RNA were used for cDNA synthesis. Primers flanking the minigene were designed for PCR amplification, and the transcription bands of different sizes were detected by agarose gel electrophoresis. Bands of varying sizes were then sent for sequencing validation.


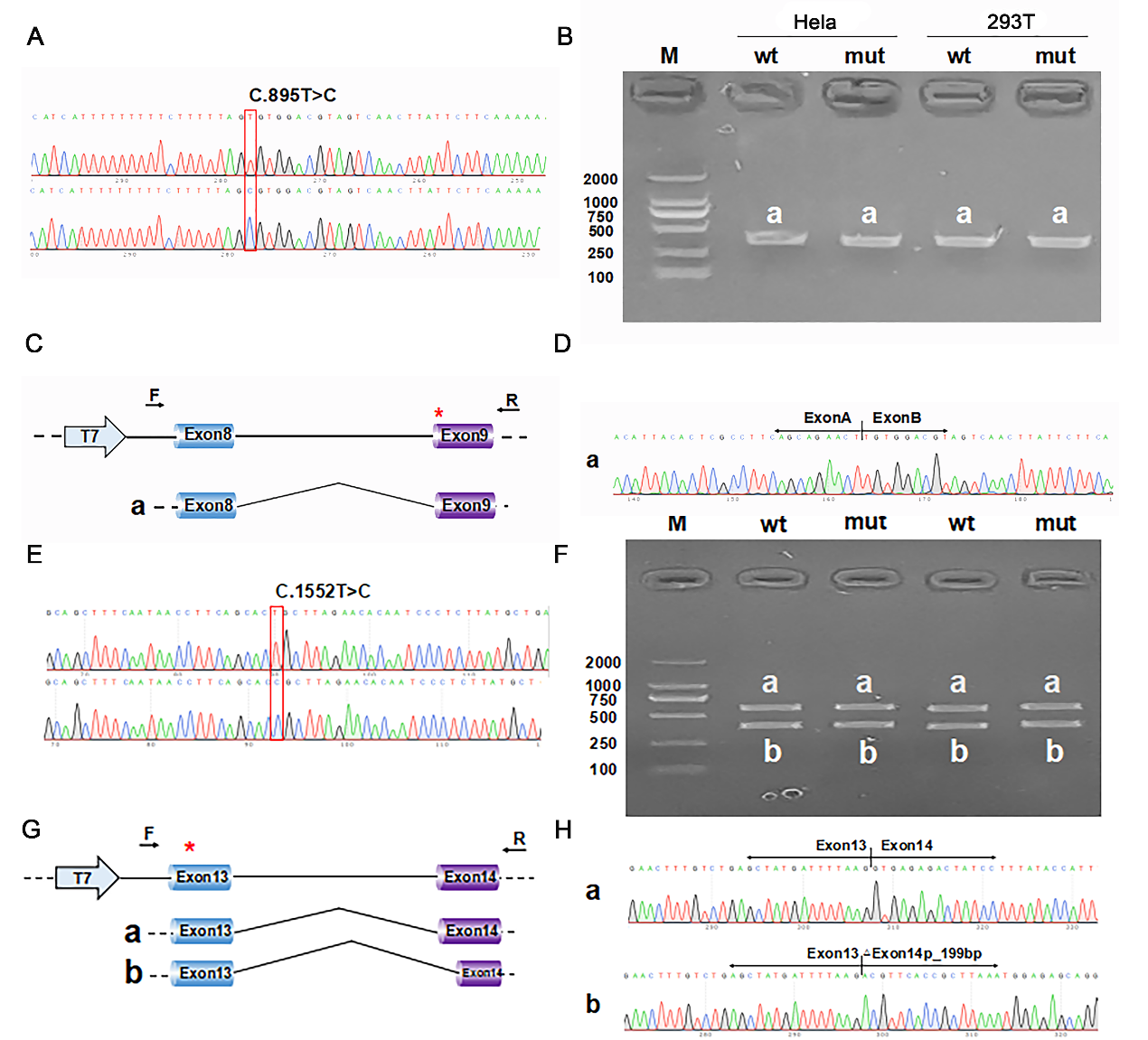
 Supplementary Figure 1. c.895T>C Minigene Results. A: Sequencing diagram of pcDNA3.1 vector construction; top is wt, bottom is mut; B: Gel run diagram for RT-PCR transcription analysis; bands are labeled as a in MCF-7 and 293T cells; C: Construction strategy and splicing schematic of minigene; D: Sequencing result diagram corresponding to the spliced bands. Red * indicates the mutation position. E: Sequencing diagram of pcDNA3.1 vector construction; top is wt, bottom is mut; F: Gel run diagram for RT-PCR transcription analysis; bands are labeled as a in MCF-7 and 293T cells; G: Construction strategy and splicing schematic of minigene; H: Sequencing result diagram corresponding to the spliced bands.
